# Physicochemical Properties of Lutein-Loaded Microcapsules and Their Uptake via Caco-2 Monolayers

**DOI:** 10.3390/molecules23071805

**Published:** 2018-07-20

**Authors:** Tong Zhao, Fuguo Liu, Xiang Duan, Chunxia Xiao, Xuebo Liu

**Affiliations:** Laboratory of Functional Chemistry and Nutrition of Food, College of Food Science and Engineering, Northwest A&F University, Yangling 712100, China; tongz@nwafu.edu.cn (T.Z.); xiangduan@nwafu.edu.cn (X.D.); chunxiaxiao@nwsuaf.edu.cn (C.X.)

**Keywords:** lutein, microcapsules, dispersibility, storage stability, cell absorption

## Abstract

Lutein is one of the most important carotenoids that can be utilized in foods as a natural pigment and nutraceutical ingredient to improve eye health. However, its utilization is limited due to its poor solubility. Chemically, the highly unsaturated structure of lutein makes it extremely susceptible to light, oxygen, heat, and pro-oxidants and therefore easily oxidized, decomposed or dissociated. In this study, we aimed to imbed natural lutein to improve its storage stability and enhance its water dispersibility. As two commonly studied water-soluble and water-insoluble food-grade surfactants, sodium caseinate (NaCas) and lecithin were chosen as the wall materials, and lutein-loaded NaCas microcapsules and lecithin microcapsules were prepared, the results revealed the lutein-loaded NaCas microcapsules not only exhibited better dispersibility and stability than those of lutein-loaded lecithin microcapsules, but also were more stable when stored at 4 °C, 25 °C, 37 °C. Moreover, the lutein-loaded NaCas microcapsules were more easily absorbed by the intestinal Caco-2 cells than natural lutein. Considering the dispersibility, stability and cell absorption effect, the NaCas-based microparticle is a potential carrier for lutein.

## 1. Introduction

Lutein, a xanthophyll (hydroxylated carotenoid), is one of the most important carotenoids, and usually is found in appreciable levels in green leafy vegetables and eggs [1,2,3]. Like other carotenoids, lutein is usually utilized as a natural pigment from plants. It is a well-known macular pigment, along with zeaxanthin; deriving this name due to the fact that they are found in higher amounts in the macula (central portion of the retina) [4]. Lutein has a very high economic value according to a 2015 report, which said that lutein market was the fastest growing market among the carotenoids with a market value of around US $233 million in 2010, projected to grow to US $309 million by the end of 2018 [5].

As a natural antioxidant, lutein had a protective effect against oxidative damage of egg yolk lecithin liposomal membranes induced by exposure to UV radiation and incubation [6]. Some epidemiologic studies revealed that lutein was able to prevent age-related cataracts and macular degeneration from the development of these two common eye diseases [7]. It may absorb the blue light that reaches the eyes and is supposed to act as antioxidants by scavenging free radicals oxygen or quenching singlet oxygen, because of the accumulation of xanthophyll in the pigment region of eyes, and decrease the oxidative stress in the retina [2,8,9]. In order to intake an appropriate amount of lutein in daily life, the Joint FDA/WHO Expert Committee on Food Additives (JECFA) gave a recommended daily intake dose of 0–2 mg/kg body weight per day for lutein and zeaxanthin [10]. Additionally, in 2014, a suggested exact intake dosage of lutein for human has been reported, 10 mg/day has been proven to be an effective dose for providing protection against disease such as age-related macular degeneration and cataracts [11]. Dosages of up to 40 mg/day in humans showed no adverse effects after eye examinations, but excessive lutein intake may have potential effect on metabolism burden of some organs, e.g., liver [12].

Unfortunately, humans are unable to synthesize lutein in vivo and the oral bioavailability of lutein is limited because of its poor water-solubility [4]. Besides, one of the other main challenges while utilizing lutein is the poor chemical stability since it is unstable to oxygen, heat and light due to lutein’s eight conjugated double bond structure. Not only can the poor stability of lutein exposed to outside environment lead to a loss of bright yellow color and bioactivity, but also can lead to loss of product quality and poor consumer acceptance [13]. In order to increase the physicochemical stability, bioavailability and solubility of lutein in aqueous media, a lot of efforts have been paid utilizing various approaches such as self-nanoemulsifying drug delivery systems (SNEDDS) technology [13,14,15], liposomal delivery [16,17,18], nanoemulsion [13,19], nanocapsules [20], encapsulation with polysaccharide [21,22,23,24,25,26].

Microencapsulation is widely utilized for masking undesirable odors and flavors in dry powder from finished products and for improving the stability of oil and fatty acid against oxidation since the 1940s. In general, the size of microencapsulation ranges from 1 μm to 1000 μm, and it is defined as a microsphere with an active core surrounded by a coating of wall material. Normally, the composition of the core is one or several ingredients and the wall is single, double and multi-layered [27]. In the case of the wall agent, it can be made up of hydrophobic and hydrophilic material [28,29]. In this study, we aimed at finding useful food-grade agents as lutein carriers to enhance water-dispersibility, storage stability and bioavailability. According to centrifugal sedimentation technique, the physicochemical properties, including micro-morphology, water-dispersibility and encapsulation efficiency were assessed, and the effect of temperature, lightness and pH on the lutein retention rate was investigated. Additionally, the in vitro release of lutein from the microspheres and its uptake utilizing an in vitro Caco-2 cell culture model were evaluated, which can be utilized as a simple, inexpensive, and reproducible tool to collect preliminary information on its bioavailability. The results of this research may lead to novel food-grade colloidal delivery systems suitable for incorporating lutein into food, supplement, or pharmaceutical products.

## 2. Results and Discussion

### 2.1. Encapsulation Efficiency of Lutein in the Microcapsules

The encapsulation efficiency of lutein in two different microcapsules was measured by UV-Vis spectroscopy and it was 89.95% and 81.24% in NaCas and lecithin particles, respectively. The wall material of NaCas tended to wrap more lutein than lecithin. It was deduced that since the spray-drying was the main microcapsule preparation procedure, and the temperature of this drying process was around 180 °C, which is 20 °C higher than the melting point of lecithin, therefore, lecithin was inclined to melt and a number of lutein molecules tended to be lost during this period.

### 2.2. Water Dispersibility and Microstructure of Lutein and Lutein-Loaded Microcapsules

**Figure 1:**
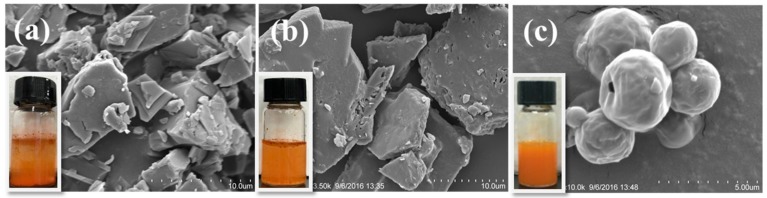
Appearance (dispersed in deionized water at a lutein concentration of 1 mg/mL) and SEM morphology images of native lutein (**a**); lutein-loaded-lecithin microcapsules (**b**) and lutein-loaded NaCas microcapsules (**c**).

In order to investigate the dispersibility improvement of lutein, the appearance of lutein and other sample powders was compared (Figure 1). At the same lutein concentration (1 mg/mL), different sample powders were dispersed in 3 mL deionized water, respectively (the lutein concentration of each sample was valued by HPLC, and the results are as follows: lutein-loaded lecithin microcapsules: 2.740 ± 0.1 mg/mL, lutein-loaded lecithin/NaCas microcapsules: 2.138 ± 0.4 mg/mL, lutein-loaded NaCas microcapsules: 2.385 ± 0.04 mg/mL). Natural lutein and lutein-loaded lecithin microcapsules appeared as insoluble particulates because of the poor water solubility. While the homogeneous dispersions could be obtained from lutein-loaded NaCas microcapsules, which suggests that NaCas, a hydrophilic agent, does contribute to dissolve insoluble matter in deionized water. It was reported that κ-caseins exhibited the best hydrophilic property among four types (α_s1_-, α_s2_-, β- and κ-caseins) of caseinates [30].

The morphological aspects of lutein and lutein-loaded microcapsules were investigated via scanning electronic microscopy as shown in Figure 1. Natural lutein and the lutein-loaded lecithin microcapsules showed irregular appearances with a hardened shapes, while the lutein-loaded NaCas microsphere showed spherical microcapsules indicating a proper encapsulation. The average size range of the particles was found to be in the range of 2–4 μm.

### 2.3. Storage Stability of Lutein and Lutein-Loaded Microcapsules

#### 2.3.1. Impact of Temperature

**Figure 2:**
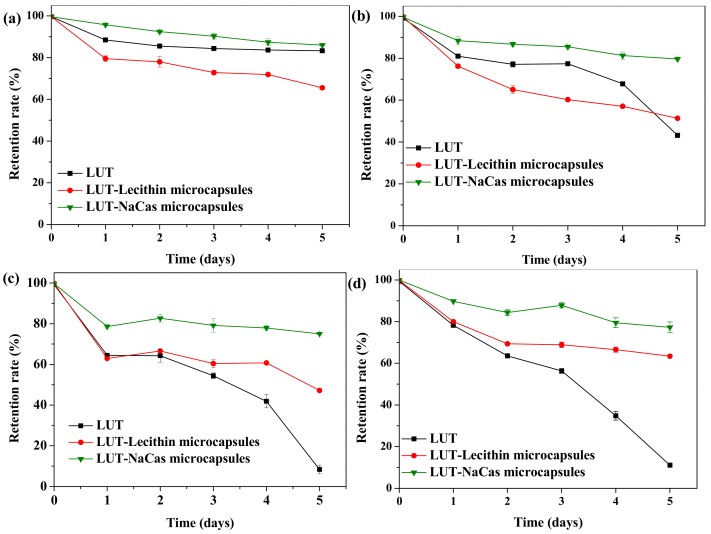
Effect of storage conditions on retention rate of lutein (LUT) in its native form and in microcapsules, (**a**) 4 °C in dark, (**b**) 25 °C in dark, (**c**) 37 °C in dark (**d**) 25 °C in bright sunshine. Data points represent means (n = 3) ± standard deviations. Some error bars lie within the data points.

The stability of microcapsules during manufacture, transport, storage and utilization is critical for their practical application [13]. Any changes in storage temperature may influence the stability of particles through numerous mechanisms. High temperature causes conformational changes of any emulsifier molecules adsorbed to the droplet surfaces, which is able to alter their ability to stabilize the droplets against aggregation [31]. However, this effect is less important for flexible proteins such as sodium caseinate. From Figure 2 it can be seen after storage for 5 days, the retention rate of lutein in natural lutein crystal and lutein-loaded NaCas powders was over 80% (82.90% for lutein and 86.01% for lutein-loaded NaCas powders), while its retention rate in lutein-loaded lecithin microcapsules was 65.13% at 4 °C (Figure 2a). In addition, the lutein retention rate of natural lutein crystal was declined dramatically to 43.19% on the fifth day at 25 °C and dropped to 8.36% at 37 °C (Figure 2b,c), while its retention rate in lutein-loaded NaCas powder was 79.83% at 25 °C (Figure 2b); and 75.02% at 37 °C on the fifth day (Figure 2c). It could be found that the redispersion of lutein-NaCas microcapsules was relatively stable to thermal processing. Different treatments showed significant differences on retention rate (Figure 2). The research by Darewicz and O’Kennedy [32,33]. suggested sodium caseinate was a pretty flexible and disordered protein, and therefore it was less likely to change through hydrophobic attraction or disulfide bonds. Yet, lutein-loaded lecithin powders exhibited poor storage stability, indicating that the egg yolk lecithin are inefficient emulsifiers for lutein encapsulation due to the changes in membrane composition during storage [34].

#### 2.3.2. Impact of Lightness

As we know the lutein is a kind of carotenoids which is highly susceptible to light, oxygen, and it is readily decomposed. Therefore, in order to investigate the impact of light on the stability of lutein during storage, samples were placed in test tubes and stored at room temperature with samples exposed to daylight. The lutein retention rate was measured every day through a 5 day test. According to Figure 2d it is easily found that the retention of lutein in natural lutein crystals showed a declining trend before it reached a lowest point at 11.07% on the fifth day, while lutein-loaded lecithin, lutein-loaded NaCas microcapsules showed different decline trends on the first five days, on the fifth day they presented a retention rate of lutein with 66.37% and 78.27%, respectively.

According to the above results, it can be found that lutein-loaded NaCas microcapsule has showed the best stability, a relatively high water dispersibility and a spherical micromorphology among lutein crystal, lutein-loaded lecithin microcapsules, and lutein-loaded NaCas microcapsules. Therefore, in the following experiments this study mainly measured other physicochemical properties and biological properties between lutein crystal and lutein-loaded NaCas microcapsules.

#### 2.3.3. Impact of pH

According to the previous studies [35,36], the protein-coated lipid droplets are mainly stabilized by electrostatic repulsion, and therefore they are highly susceptible to pH changes in solution. Thus, in this study we intended to figure out the impact of pH on the stability of lutein and lutein-loaded NaCas microcapsules. Based on the pH environment of humans’ main digestive organs (oral cavity: pH 6.5~7.5; stomach: pH 1.5~4.0; intestines: pH 4.0~7.0), pH values of 2, 5 and 7 were tested.

**Figure 3:**
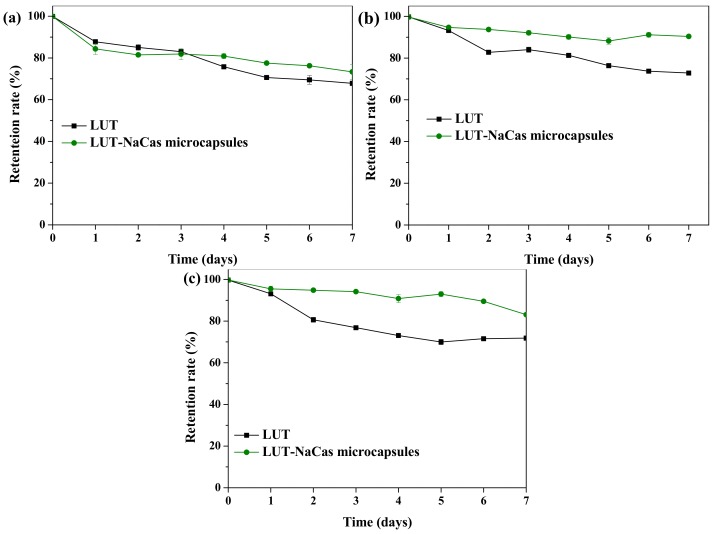
Effect of storage pH values (**a**) pH 2; (**b**) pH 5; (**c**) pH 7 on the retention rate of lutein (LUT) in its natural form and lutein-enriched microcapsules stabilized by sodium caseinate after 7 days of storage. Data points represent means (n = 3) ± standard deviations. Some error bars lie within the data points.

From the results (Figure 3) it can be found after being redispersed at pH 5 and pH 7 lutein-loaded NaCas microparticles were more stable than natural lutein crystals. As a result, it was speculated that the lutein-loaded protein droplets were fairly stable to aggregation with no statistical difference at pH values far below or above their isoelectric point.

### 2.4. FTIR Spectroscopy and DSC

**Figure 4:**
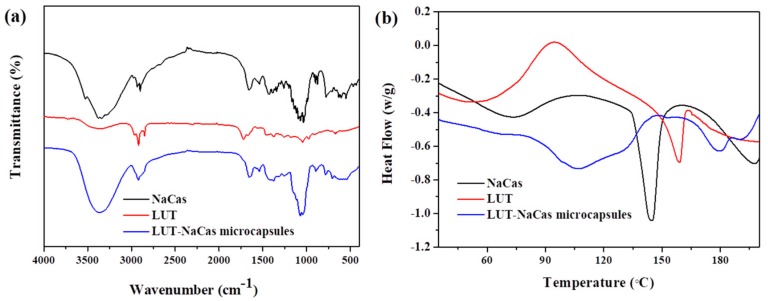
FTIR spectra (**a**) and DSC thermograms (**b**) of the native sodium caseinate (black), lutein crystals (red) and encapsulated lutein (blue).

The characteristic transmittance of lutein crystal, NaCas and lutein-loaded NaCas microcapsules were measured by FTIR spectroscopy (Figure 4a). The protein bands of the amide I (1600–1700 cm^−1^) and amide II (1510–1530 cm^−1^) bands dominated the spectra. The strong transmittance of lutein was at 3415.82 cm^−1^, which was the O-H stretching vibrations mode, while this strongest transmittance shifted to 3413.90 cm^−1^ after encapsulated by NaCas, and the strongest transmittance of NaCas was at 3415.82 cm^−1^. The strong transmittance of lutein, NaCas and lutein-loaded NaCas microcapsules were at 1620.15 cm^−1^, 1618.23 cm^−1^ and 1639.44 cm^−1^ respectively. Interestingly, none of most bands of lutein were observed in the spectra of the lutein-loaded NaCas microcapsules, and there was no obvious difference in the FTIR spectra between NaCas and lutein-loaded NaCas microcapsules, implying that the entrapping procedure did not cause an obvious effect on the structural conformation of the proteins and this effect may due to the relatively low concentration of nutraceuticals or because of their interactions with the surrounding matrix [37], the encapsulation may have altered their electromagnetic absorption characteristics of lutein.

DSC profiles of the lutein, NaCas and lutein-loaded NaCas microcapsules over a broad range of temperatures (from 35 °C to 200 °C) are shown in Figure 4b. The DSC curve of lutein exhibited a sharp peak at 159.1 °C corresponding to the melting point of lutein crystals. The microcapsules samples of lutein-loaded NaCas powder had a melting point at 179.7 °C, which indicated that lutein was not crystalline in the microcapsules but rather it was amorphous. The NaCas powder sample had a melting temperature of 144.7 °C. In addition, the glass transition temperature was higher in lutein-loaded NaCas microcapsules (107.1 °C) than in lutein crystal (51.5 °C) and NaCas (73.4 °C). The different T_g_ results showed that the mixture powders samples had a relative high thermal construction stability than lutein crystals’ [38].

### 2.5. In Vitro Release Study of the Lutein-Loaded NaCas Microcapsules

**Figure 5:**
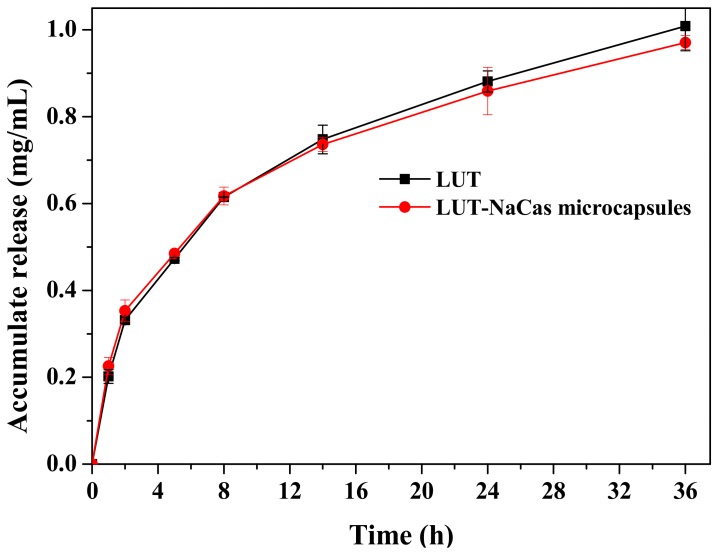
The in vitro release of lutein (black) and lutein-loaded NaCas microcapsules (red) from dialysis bag (MW: 8000–14,000 kDa) at 37 °C during a 36 h release.

The lutein release behaviors from lutein crystal and the lutein-loaded microspheres microcapsules were investigated via in vitro release study. According to Figure 5, during the first 5 h, the lutein-loaded NaCas microcapsules released quickly from the dialysis tube in the shaker at 37 °C. The release profile of lutein from raw lutein was similar as that of the microencapsulated lutein. However, the release rates of lutein and lutein-loaded NaCas microcapsules began to show release differences after 14 h and the natural lutein started to release faster compared with the microsphere after then. Natural lutein had an accumulated release of 1.01 μg/mL at the 36 h point, while the lutein-enriched microsphere had an accumulated lutein release of 0.97 μg/mL. The mechanism of lutein release from the microcapsules might be controlled by swelling/erosion of wall material. Water molecules can penetrate into the matrix of the microcapsules leading to its swelling as the water diffuses from the outer solution into the matrix, the meshes of the polymeric network become increasing wider [39], allowing the lutein to diffuse into the outer aqueous environment. From then on, the release rate became slower than the initial 2 h, which was probably because of the long diffusion route of lutein entrapped deeply in the wall materials [40].

### 2.6. Absorption of Lutein in Caco-2 Cell Monolayers

**Figure 6:**
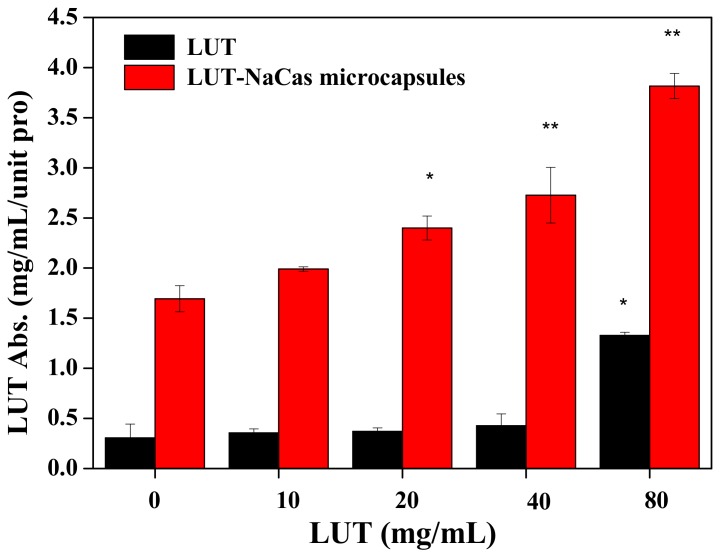
The in vitro absorption of lutein (LUT) and lutein-loaded NaCas microparticles in Caco-2 cells with different concentrations (0, 10, 20, 40, and 80 μg mL^−1^) after incubation for 12 h at 37 °C. Data are expressed as the mean ± SD (n = 3). Within the same samples, values with * are significantly different (*p* < 0.05), values with ** are significantly different (*p* < 0.01).

The dose-response absorption of lutein in Caco-2 cells can be utilized as a simple, inexpensive, and reproducible tool to collect preliminary information on its bioavailability. Therefore, a content of 0, 10, 20, 40, 80 μg/mL lutein in raw lutein and lutein-loaded NaCas microcapsules were chosen to treat cells for 12 h. The lutein content in extracellular cells it was assessed by HPLC. Lutein content in lutein crystal group had a gradual increase when adding the lutein solution from 0–80 μg/mL, while in intracellular the lutein content rocketed to 1.33 μg/mL/unit protein. Meanwhile, the lutein content in intracellular cells in lutein-loaded microparticles group had a more stable gradual increase from 1.99 to 3.82 μg/mL/unit pro, while in extracellular the lutein content increased faster from 0.36 to 10.17 μg/mL/unit pro (Figure 6). The result shows that the lutein-loaded NaCas microcapsules are more easily absorbed by the intestinal cells than natural lutein. Based on all aforementioned results, the improved stability and absorption of lutein may be affected by the thick coverage of NaCas interface in the microcapsule. More work should be done in detail on the structural changes of NaCas in the dispersions.

## 3. Materials and Methods

### 3.1. Materials

Lutein was purchased from Xi’an Day Natural Inc. (Xi’an, China). The lutein product had a purity of 80% (*w*/*w*) according to the vendor. Sodium caseinate (NaCas) was purchased from MG (Melbourne, VIC, Australia), egg yolk lecithin (the content of phosphatidylcholine is around 80%, and phospholipids is approximately 3.7%.) was purchased from Shanghai East China Normal University chemical plant (Shanghai, China), Na_2_HPO_4_·12H_2_O, NaCl, KCl, KH_2_PO_4_, acetone and all other reagents utilized were of analytical grade and purchased from Zhiyuan Chemical Co., (Tianjin, China).

### 3.2. Preparation of Lutein-Loaded Microcapsules

Since previous studies [41,42] reported that lecithin and NaCas had suitable behavior in encapsulation, different formulations containing lecithin or NaCas were designed. According to preliminary experimental results, 2.25% (*w*/*w*) of lecithin or NaCas and 0.5% (*w*/*w*) lutein were preferred to make the microcapsules. The lutein-loaded microsphere was prepared as follows: firstly, 18 g of NaCas (or 18 g lecithin) was dissolved in 600 mL of 40% (*v*/*v*) aqueous ethanol, then they were mixed by a magnetic stirrer and heated at 60 °C for 5 min until the wall materials dissolved completely. Meanwhile, 4 g of lutein was dispersed in 200 mL of 40% ethanol, stirred until no insoluble particles existed. Then the lutein solution was added into the wall material solution drop-by-drop and the mixture was stirred continuously until they mixed well. After that, the mixture was filtered to remove undissolved large particles by a 100 mesh sieve before blending them at 10,000 rpm for 4 min utilizing a high-speed homogenizer (T25, IKA, Staufen, Germany). The homogenizer was equipped with a 13 mm diameter rotor-stator and the range of rotation speed was set from 2800 to 24,000 rpm. In order to avoid over-heat of homogenizer during employing, it was taken a 15 s break after every half minute utilization. The lutein-loaded NaCas or lecithin emulsion was finally spray-dried at an inlet temperature of 180 °C and an outlet temperature of 90 °C by a SP-1500 spray-dryer (ShangHai SunYi Tech Co. Ltd., Shanghai, China).

### 3.3. Encapsulation Efficiency

The lutein encapsulation efficiency (EE, %) was calculated utilizing the following equation:

(1) $$\mathrm{Encapsulation}\;\mathrm{Efficiency}\left(\%\right)=\left(1-\frac{\mathrm{un}-\mathrm{entrapped}\;\mathrm{lutein}}{\mathrm{total}\;\mathrm{lutein}}\right)\times 100$$

To measure the total lutein, 100 mg lutein was dispersed in 5 mL deionized water; the particles were dispersed completely with the aid of ultrasonic machine (KQ-5000DE, Shumei, KunShan, China). Then the lutein dispersions were transferred into a 100 mL brown flask, and 95 mL acetone was added and the dispersions was vortexed for 2 min, then the absorbance at 446 nm wavelength was measured using a UV/VIS spectrophotometer (model Mini UV 1240, Shimadzu, Tokyo, Japan). Acetone was utilized as a blank; the measurement was repeated for three times. The lutein content (%) was calculated utilizing the following equation:

(2) $$\mathrm{Lutein}\;\mathrm{Content}\left(\%\right)=\frac{A\times \mathrm{dilution}\;\mathrm{times}}{2540\times G}\times 100$$

where, A stands for the values in maximum UV absorption wavelength at 446 nm; G stands for the weight of sample powder (g); 2540 is the lutein extinction coefficient in acetone.

To measure the unentrapped lutein, 0.2 g sample microcapsules was weighed in 50 mL centrifuge tube before a 20 mL acetone was added and shook for 1 min. Then it was centrifuged it under a condition of 6000 rpm/min and 4 °C for 5 min. 5 mL supernatant was transferred into a 10 mL centrifuge tube, and dried completely by a nitrogen blow-drying apparatus (QF-3800 Qimei, Tianjin, China). After that the dry film was dissolved and diluted it for 4 times with acetone to measure its absorbance under 446 nm wavelength by the UV/VIS spectrophotometer.

### 3.4. Scanning Electron Microscopy (SEM)

SEM (S-3400N; Hitachi, Japan) was utilized to determine the appearance of natural lutein crystal, lutein-loaded lecithin powder and lutein-loaded NaCas powder. The acceleration voltage was 5 kV [43]. Each sample was mounted with a two-side adhesive tape on brass stubs and then sputtered with platinum particles. These platinum coated particles were examined under SEM for surface morphological analysis.

### 3.5. Stability of Lutein Microcapsules

Based on the ordinary storage temperature, and pH values of human digestive system, the sample powders were stored for a week at different storage temperatures (4 °C, 25 °C, 37 °C) and different pH values (2, 5, 7) respectively, and the lutein content of these samples were calculated by equation 2. The pH values were adjusted to the desired values utilizing 1 M hydrochloric acid or sodium hydroxide solutions. The samples of different pH values were stored at 4 °C. The lutein retention rate was calculated by equation (3):

(3) $$\mathrm{Lutein}\;\mathrm{Retention}\;\mathrm{Rate}\left(\%\right)=\frac{\mathrm{residual}\;\mathrm{lutein}\;\mathrm{content}}{\mathrm{initial}\;\mathrm{lutein}\;\mathrm{content}}\times 100$$

#### 3.5.1. Impact of Lightness

10 mg lutein, lutein-loaded lecithin microcapsules, and lutein-loaded NaCas microcapsule powder was respectively dispersed with 5% ethanol solution in centrifuge tubes. Samples were then stored at room temperature with bright sunshine for 7 days. The content of lutein in different samples was measured according to the aforementioned method.

#### 3.5.2. Impact of Temperature

10 mg lutein and lutein microcapsules samples were dissolved in ethanol solution (5%) and stored at 4 °C, 25 °C and 37 °C separately in the dark for 5 days. Total lutein content was measured as the aforementioned method during the storage.

#### 3.5.3. Impact of pH

Initially 10 mg lutein crystal and lutein microcapsules samples were dissolved in ethanol solution (5%) separately, in order to simulate the human digestive environment, these two solutions were adjusted to pH 2, 5, 7 utilizing 1 M HCl or 1 M NaOH. The samples were stored in the dark at 4 °C for 7 days. The content of lutein in different samples was measured according to the aforementioned method.

### 3.6. Fourier Transform Near-Infrared Spectroscopy (FTIR)

The FTIR spectra were recorded by Fourier Transform Near-infrared Spectrometer (VERTEX-80, Bruker, Ettlingen, Germany). To prepare the sample tablets, 1 mg sample powder and 30 mg dried KBr powder was mixed completely, and then it was pressed into a thin tablet with a pressure of 10 MPa by the tablet machine. The spectral region was set from 400 to 4000 cm^−1^.

### 3.7. Differential Scanning Calorimetry (DSC)

DSC analysis of powder samples of lutein and lutein-loaded microcapsules were characterized utilizing a model Q2000 calorimeter (TA Instrument, New Castle, DE, USA). 3 mg of powdered sample was sealed in hermetic aluminum pans and then heated from 30 °C to 250 °C at a rate of 5 °C/min. Nitrogen was utilized as the transfer gas at a pressure of 0.5 MPa.

### 3.8. Evaluation of In Vitro Release from Dialysis Bag

The studies were carried out to characterize the in vitro release of lutein from microcapsules at 37 °C utilizing a dialysis bag (8000–14,000 kDa Solarbio, Beijing, China) and glass vessels (100 mL). A certain weight of powder was dissolved (the content of lutein is 0.2 mg/mL) in a dialysis bag filled with 10 mL phosphate buffer solution (pH 7.4–7.6). Then the dialysis bag was emerged in a 100 mL glass vessel filled with PBS and incubated in a shaker (80 rpm/min) at the temperature of 37 °C. 1 mL dispersion was sampled for assays at regular intervals (n = 3). The remaining dispersion was supplemented with a 1mL of the fresh phosphate buffer solution to continue the test. The released concentration of lutein was measured by HPLC (LC-15C, Shimadzu, Kyoto, Japan) with chromatographic column C18 (Agilent, Zorbax Eclipse XDB-C18, Waldbronn, Germany), according to the standard curve from previous reference [44].

### 3.9. Absorption of Lutein in Caco-2 Cell Monolayers

Caco-2 cells were maintained in advanced DMEM-high glucose nutrient medium (HyClone, Beijing, China), supplemented with 15% fetal bovine serum (Gemini, Woodland, CA) and 1% penicillin-streptomycin (Beyotime Biotechnology, Shanghai, China) in a humidified incubator (Thermo Fisher Scientific, Waltham, MA, USA) at 37 °C and 5% CO_2_. Cells between the 6th to 8th passages were utilized for the experiment [45]. The uptake and secretion of lutein was tested utilizing the two delivery vehicles: (i) lutein crystal, (ii) lutein-loaded NaCas microcapsules. Cells were maintained in medium supplemented with 1% antibiotics (penicillin-streptomycin) and seeded at petri dish for 6 days to attain the complete morphology. The cell cytosol was treated with a range of lutein content (0, 10, 20, 40, 80 mg/mL) as the Caco-2 cell grew to 90%. After being treated for 24 h, the cell cytosol was collected in a centrifuge tube, stored at −80 °C before analysis by HPLC. Protein content in the cell lysate was determined utilizing a BCA kit (Thermo Fisher Scientific, Rockford, IL, USA) to reflect cell count and utilized for standardization of lutein content.

As for the beginning of the treatment for the Caco-2 cells, the apical side of cells was washed twice by phosphate buffer solution to remove any serum containing media and lutein enriched serum-free media. The basolateral compartment was filled with serum-free media. Lutein in the medium was stable in the culturing condition for over 24 h. In the dose-response experiment, a 24 h incubation time was employed to examine the effect of vehicle on lutein uptake and was selected based on the lutein concentration. At the end of the incubation time, apical media were collected. Cells remaining on the permeable filters were washed twice with phosphate buffer solution, and the lysed with 120 μL cell lysis buffer (Beyotime, Biotechnology) and 20 μL DMSO for 10 min at 4 °C to detach cells from the dish. Then centrifuged at 5000 rpm, 1 min, 4 °C, the supernatant was collected in 1.5 mL centrifuge tubes, then 5 μL supernatant was ingested and diluted for 3 times to measure cell protein with BCA kit. The rest of supernatant was filtered by 0.45 μm organic filters (Jinlong, Tianjin, China). Those unfiltered samples were stored at −80 °C before HPLC analysis.

### 3.10. Statistical Analysis

All experiments were conducted in 3 replications. Data were subjected to analysis of variance (ANOVA) on SPSS 18.0 for Windows (IBM Inc., Armonk, NY, USA). Differences between means were evaluated by Duncan methods at the significance level of 5%.

## 4. Conclusions

In summary, different lutein-enriched microcapsules (lutein-loaded lecithin microcapsules and lutein-loaded NaCas) were prepared. Through investigations on the effect of temperature, light and pH it was found that NaCas showed better abilities to stabilize lutein crystals against light and heat. Besides, this lutein-loaded NaCas microencapsulates had a better water-dispersibility, stability and cellular uptake in vitro than native lutein. These results with lutein-enriched microcapsules have potential applications, which can not only facilitate the rational design and fabrication of microencapsulation delivery system for lutein utilization in functional foods and functional beverages, but also have implications for future lutein studies.
